# Vitamin B and Vitamin C Affect DNA Methylation and Amino Acid Metabolism in *Mycobacterium bovis* BCG

**DOI:** 10.3389/fmicb.2020.00812

**Published:** 2020-04-22

**Authors:** Ningning Song, Yongqiang Zhu, Yingying Cui, Mingyue Lv, Yiyi Tang, Ziyin Cui, Guanghui Dang, Huajun Zheng, Siguo Liu

**Affiliations:** ^1^State Key Laboratory of Veterinary Biotechnology, Harbin Veterinary Research Institute, Chinese Academy of Agricultural Sciences, Harbin, China; ^2^Shanghai-MOST Key Laboratory of Health and Disease Genomics, Chinese National Human Genome Center at Shanghai, Shanghai, China

**Keywords:** vitamin, methylation, amino acid, *Mycobacterium*, cysterine synthase A

## Abstract

Vitamins are essential nutrients and key cofactors of enzymes that regulate cellular metabolism, and also activate the immune system. Recent studies have shown that vitamin B1 (V_B__1_) and vitamin C (Vc) can inhibit *Mycobacterium tuberculosis* growth, but the precise mechanism is still not well understood. In the present study, we have used RNA-sequencing (RNA-seq), liquid chromatography coupled to mass spectrometry (LC-MS) and single-molecule real-time (SMRT) sequencing to analyze the transcriptional, metabolic and methylation profiles of *Mycobacterium bovis* BCG when treated with V_B__1_ and Vc. Our results show that, after vitamin treatment, variant metabolites were mainly clustered in pathways related to amino acid metabolism. Treatment with both vitamins significantly up-regulated the gene encoding cysteine synthase A. Additionally, only BCG that was treated with V_C_ showed m4c modifications. Genes harboring this methylation were up-regulated, suggesting that m4c methylation can promote gene transcription to some extent. Overall, this study contributes to the understanding of the effects of V_B__1_ and V_C_, and suggests that these vitamins constitute potential anti-tuberculosis drugs.

## Introduction

*Mycobacterium tuberculosis* (Mtb) is a well-known intracellular pathogen which causes almost nine million cases of tuberculosis every year and latently infects up to a third of the world population (World Health Organization [WHO], 2019). Mtb is able to persist in the host for decades without causing clinical symptoms, and has evolved adaptation mechanisms to survive in the harsh environment of the host, such as hypoxia, nutrient deprivation or low pH (Betts et al., 2002; Fisher et al., 2002; Schnappinger et al., 2003). This adaptation is possible by regulation of gene expression, which in turn can be controlled by DNA methylation (Shell et al., 2013).

DNA methylation has been known since the 1950s (Luria and Human, 1952). In prokaryotes, it represents a primitive defense mechanism against phages and viruses, known as the Restriction-Modification (RM) system. Although adenine is the most important methylation target, e.g., *N*^6^-methyl-adenosine (m6A), there are other two types of methylation. These are found in cytosine and depend on the modification site: m4C (*N*^4^-methyl-cytosine) and m5C (*C*^5^-methyl-cytosine) (Murphy et al., 2013). In general, m6A has been used as the main signal for epigenetic regulation in bacteria, m5C has been mainly described in mammals and plant studies (Kim et al., 2009), whereas m4C is mainly present in bacteria (Kumar et al., 2018).

DNA methylation plays an important role in the epigenetic regulation of eukaryotes via gene expression modulation (Moore et al., 2013). Indeed, methylation of promoter and gene body can either silence or promote expression, respectively (Yang et al., 2014). In bacteria, DNA methylation may also play regulatory roles in the control of DNA replication or transcription (Reisenauer et al., 1999), and this may contribute to environmental adaptation and metabolic activity (Blow et al., 2016).

Single-molecule real-time (SMRT) sequencing (Pacific Biosciences) is a recently developed DNA sequencing method where kinetic data can also be used to distinguish base modifications, e.g., methylation, at the single nucleotide level (Flusberg et al., 2010). Several bacterial methylomes have been determined using SMRT sequencing technology (Fang et al., 2012; Krebes et al., 2014; Zhu et al., 2016). Specifically, a methylome analysis of *M. tuberculosis* complex (MTBC) was reported by Zhu et al. (2016).

Vitamins are organic compounds that cannot be produced by the host organism. Even when they are produced, concentrations are low and external supplementation from the diet or from commensal bacteria is required. Vitamins have been found to regulate immunity, and a number of studies have reported the effect of vitamins as adjunct to treat tuberculosis (Parida et al., 2015; He et al., 2018). For example, vitamins A, C and D have been used as adjunct to complement anti-tuberculosis drugs (Dini and Bianchi, 2012; Vilcheze et al., 2013; Syal et al., 2015). The active form of thiamin (Vitamin B1, V_B__1_), thiamin diphosphate (ThDP) (Begley et al., 1999; Settembre et al., 2003; Pohl et al., 2004), is an essential cofactor in all organisms, taking an important role in energy metabolism and degradation of sugars. In addition, V_B__1_ plays an important role in the activation of the immune system, nerve tissue repair, neuronal communication and cell-membrane dynamics (Gibson and Blass, 2007; Manzetti et al., 2014). Recent studies have shown the effects of V_B__1_ both *in vitro* and *in vivo*; V_B__1_ inhibits *in vitro* growth of BCG with an MIC of 8 mM, whereas it limits Mtb *in vivo* growth by regulating innate immunity in a peroxisome proliferator-activated receptor γ-dependent manner (Hu et al., 2018; Song et al., 2019). V_C_ also can hamper the growth of Mtb by a mechanism that involves the reduction of ferric to ferrous ion and subsequent production of reactive oxygen species (i.e., hydrogen peroxide, superoxide and hydroxyl radicals) through Harber-Weiss cycle and Fenton reactions (Vilcheze et al., 2013). BCG is also sensitive to Vc, with an MIC is 0.8 mM (Pei et al., 2019).

Microbial metabolomics constitutes an integrated component of systems biology. By studying the complete set of metabolites within a microorganism and monitoring the global outcome of interactions between its development processes and the environment, metabolomics can potentially provide a more accurate snap shot of the actual physiological state of the cell. Furthermore, when these data are interpreted in combination with genomics, proteomics and transcriptomics data and so on, using what is termed a systems biology approach, a more holistic understanding of these systems can be achieved. Up to now, the metabolomics has contributed to characterize Mtb in terms of metabolism, growth and replication, pathogenicity, and drug resistance, from the perspective of systems biology (Swanepoel and Loots, 2014). For example, Meissner-Roloff compared the metabolomes of a hypo- and hyper-virulent Beijing Mtb strain and subsequently identified a reduction in various metabolite markers in the relatively hyper-virulent strain (Meissner-Roloff et al., 2012). Loots used metabolomics approach to identify potentially new metabolic pathways and metabolite markers to explain many of the phenotypical characteristics associated with a katG mutation and the resulting isoniazid-resistance in Mtb (Loots du, 2014). Additionally, metabolomics was to identify individual metabolites or metabolite profiles that could be used as biomarkers of early MAP (*Mycobacterium avium* subsp. *Paratuberculosis*) infection in ruminants (De Buck et al., 2014).

However, despite transcriptomics and proteomics efforts have been studied (Shiloh et al., 2008; Mishra and Sarkar, 2015; Batra et al., 2017; Martini et al., 2019), the effect of vitamin treatment on Mtb metabolomics and gene methylation remains poorly understood. DNA methylation and transcriptional regulation are tightly related and play an important role in the epigenetics of living organisms, whereas vitamins have an anti-tuberculosis effect. These prompted us to study the methylome, transcriptome and changes in metabolism of *Mycobacterium bovis* BCG after V_B__1_ and V_C_ stimulation.

## Materials and Methods

### Bacterial Strains and Culture Conditions

*Mycobacterium bovis* BCG str. Tokyo 172 strain was grown in Middlebrook 7H9 medium Becton Dickinson, supplemented with 0.05% Tween-80 (v/v), 10% ADC (Albumin-Dextrose-Catalase, BD) and 0.2% (v/v) glycerol. When OD_600nm_ reached 0.3, V_B__1_ and V_C_ were added with final concentrations of 8 and 5 mM, respectively. This was followed by incubation at 37°C for 48 h. At least three replicates were prepared for each condition. Different concentrations (0, 1, 2, and 4 mM) of cysteine were used to test the effect for BCG growth.

### Genomic DNA Extraction and SMRT Sequencing

BCG pellets were collected by centrifugation and suspended with lysis buffer (20 mM Tris⋅Cl, pH 8.0; 2 mM sodium EDTA; 1.2% Triton^®^ X-100, 20 mg/mL lysozyme). These samples were then sonicated for 5 min and incubated at 37°C for 1 h. RNase was added to the sonicated samples and incubated for 5 min at room temperature, followed by protease K addition, according to manufacturer instructions (TIANamp Bacteria DNA Kit protocol, Tiangen Biotech, Beijing, China). The concentration of DNA samples was determined using a NanoDrop ND-2000 spectrophotometer. The extracted genomic DNA from BCG was sequenced using the Pacific Biosciences RSII DNA sequencing system (Pacific Biosciences, Menlo Park, CA, United States). A 10-kb SMRTbell library was prepared from sheared genomic DNA (>5 g) using a 10-kb template library preparation workflow, according to the manufacturer. One SMRT cell was used for each sequencing.

### Bioinformatic Analyses of SMRT Sequencing Data

*De novo* assembly of the insert reads was performed with the Hierarchical Genome Assembly Process (HGAP.3) algorithm in the SMRT Portal (version 2.2.0). Circularization was achieved by manual comparison and removal of an overlap region. The final genome was confirmed by remapping of sequence data. Promoter regions were analyzed using the Neural Network Promoter Prediction tool^1^ and PePPER^2^.

Genome-wide detection of base modification and sequence motifs was performed using the standard settings (QV of modified motifs are more than 30) in the protocol ‘RS Modification and Motif Analysis.1’ included in the SMRT Portal version 2.2.0. ‘Motif score’ = (number of detections matching motif)/(number of genome sites matching motif) × (sum of log-p value of detections matching motif) = (fraction methylated) × (sum of log-p values of matches). The SMRT Portal searches (close to maximize) through of all possible motifs, progressively testing longer motifs using a branch-and-bound search. The minimal term ‘motif score’ and ‘fraction methylated’ parameters were 40 and less than 1, respectively.

### LC-MS Based Metabolomic Analysis

The BCG strains cultured with 7H9 medium in the absence and/or presence of V_B__1_ and V_C_ were collected after 48 h incubation at 37°C. The 60 mg pellets were collected by centrifugation and the supernatant were removed, the metabolites are measured by LC-MS as described (Song et al., 2019). Collected bacteria were ground and dissolved in an aqueous methanol solution (water:methanol, 1:1, v/v) at 4°C. After membrane filtration (0.22 μm), samples were used for LC-MS detection. Separation was achieved in a Shimadzu LC-30A system equipped with an ACQUITY UPLC HSS T3 (150 × 2.1 mm, 1.8 μm, Waters) column maintained at 40°C. The temperature of the autosampler was 4°C. Gradient elution of analytes was carried out with 0.1% formic acid in water (solvent A) and acetonitrile (solvent B) at a flow rate of 0.3 mL/min. After equilibration, 5 μL of each sample were injected. Solvent B was increased linearly as follows: 2% (0–0.5 min), from 2 to 50% (0.5–9 min), from 50 to 98% (9–12 min), 98% (12–13 min), from 98 to 2% (13–14 min), and 2% (14–15 min).

The ESI-MS experiments were executed on the AB 5600+ mass spectrometer with the spray voltage of 5.50 kV and −4.50 kV in positive and negative modes, respectively. Gas1 and gas2 were both set at 50 psi and curtain gas was 35 psi. The source temperature was 500°C. The mass analyzer scanned over a m/z range of 100–1,500 for a full scan at a collision energy of 45 eV with dynamic exclusion.

### Determination of Amino Acids

The amino acids of samples treated with V_B__1_ and V_C_ were detected by HR-UPLC-MS (high resolution ultra-performance liquid chromatography-mass spectrometry) as described (Song et al., 2019). Briefly, an equimolar standard mixture of 20 amino acids (i.e., glycine, L-sarcosine, L-alanine, L-valine, L-proline, L-threonine, L-isoleucine, L-leucine, L-ornithine, L-methionine, L-histidine, L-phenylalanine, L-arginine, L-tyrosine, L-aspartic acid, L-tryptophan, 4-aminobutyric acid, L-serine, L-lysine, L-glutamic acid) was prepared with concentrations 0.2, 0.5, 1, 2, 5, 10, 20, and 50 μg/mL.

Pellets of bacteria (100 mg) were ground in liquid nitrogen, and amino acids were extracted with 1 mL HCl overnight. The supernatant was collected by centrifugation at 12,000 rpm for 5 min. After addition of 100 μL of an internal standard, isotope-labeled amino acid (Alanine −d4), the sample was dried under a moderate nitrogen gas stream. A volume of 60 μL of derivatization reagent [hydrochloric acid/n-butyl alcohol (1:3, v/v)] was added into the mixture and incubated for 15 min at 65°C, and the sample was analyzed by UPLC-MS.

Chromatographic separation was achieved with an ACQUITY UPLC system equipped with a 1.7 μm C18 column (ACQUITY UPLC BEH, 2.1 × 100 mm, Waters) at 40°C. Gradient elution of the analytes was implemented with solvent A (0.1% formic acid and 0.1% heptafluorobutyric acid in acetonitrile) and solvent B (0.1% formic acid in water), at a flow rate of 0.25 mL/min. Five μL of sample were injected following equilibration. An increasing linear gradient of solvent A (v/v) was used as follows: 5% A (0–1.5 min); 5 to 20% A (1.5–2 min); 20 to 30% A (2–7 min); 30 to 98% A (7–8.5 min); 98% A (8.5–10.5 min); 98 to 5% A (10.5–11 min) and 5% A (11–12.5 min).

The ESI source was applied in positive mode by multiple reaction monitoring (MRM). The ion source capillary voltage and cone voltage were set as 3,200 and 20 V, respectively, and the desolvation temperature was set at 380°C.

### RNA Extraction and Library Construction

Since methylation and transcriptomics are both ways of studying epigenetics and they have the close relationship, to further investigate how V_B__1_ and V_C_ treatment affect BCG growth, RNA-seq was used to compare the transcriptome of the three replicates of each BCG sample (V_B__1_-treated, V_C_-treated and control). RNA-seq was performed as described (Song et al., 2019) on three replicates of bacteria treated with V_B__1_ and V_C_. Cell pellets were lysed and homogenized by high-speed agitation in a bead mill in presence of glass beads and lysis buffer. Total RNA was extracted using RNeasy Mini Kit (Qiagen) according to the instructions with one on-column DNase I treatment (Qiagen) at 37°C for 30 min, in order to remove any contaminating genomic DNA. DNase I was removed with RNeasy mini kit, according to the clean-up procedure (Song et al., 2019). The RNA integrity number (RIN) was used to inspect RNA integrity by an Agilent Bioanalyzer 2100 (Agilent Technologies, Santa Clara, CA, United States). The concentration of RNA samples was determined with a NanoDrop ND-2000 spectrophotometer. The strand-specific library was constructed using TruSeq^®^ Stranded Total RNA Sample Preparation kit (Illumina, United States). The ribosomal RNA was removed and the RNA fragments were cleaved, whereas the first and second strand cDNA were synthesized. A single ‘A’ nucleotide was added to the 3′ ends of the blunt DNA fragments with repaired ends, and subsequently connected with adapters. The purified libraries were quantified using Qubit^®^ 2.0 Fluorometer (Life Technologies, United States) and validated by Agilent 2100 bioanalyzer (Agilent Technologies, United States), in order to measure concentration and confirm insert size. The cluster was generated by cBot, and RNA sequencing was performed using a 150 bp pair-end strategy with the Illumina Hiseq X10 platform (Illumina, United States) to generate three billion bases per sample. Thereafter, raw data was acquired, followed by pretreatment using Septk1. The read length was > 90 nt. The clean reads were mapped to the *Mycobacterium bovis* BCG str. Tokyo 172 genome using Bowtie2 (Langmead and Salzberg, 2012). Differential expression analysis was performed using edgeR package (Robinson et al., 2010). Differentially expressed genes were defined as experiencing more than a 2-fold change and a FDR (false discovery rate) of less than 0.05.

Enrichment of KEGG pathways for a given gene list was calculated using a classical hypergeometric distribution statistical comparison of a query gene list against a reference gene list. The calculated *p*-value was FDR-corrected, and the corrected *p*-value < 0.05 was used as a threshold. KEGG pathways fulfilling this condition were defined as significantly enriched in regulated genes.

The raw data was deposited in the GEO repository, under accession numbers GSE114949 (V_B__1_-treated) and GSE141513 (V_C_-treated).

### Statistical Analysis

The experiments were performed at least in biological triplicate and the results are presented as the mean ± standard deviation (SD). Values were considered to be statistically significant when *P*-value was <0.05 and the statistical significance of the observed differences was assessed with one way analysis of variance (one-way ANOVA).

## Results

### DNA Methylome Analysis

**FIGURE 1:**
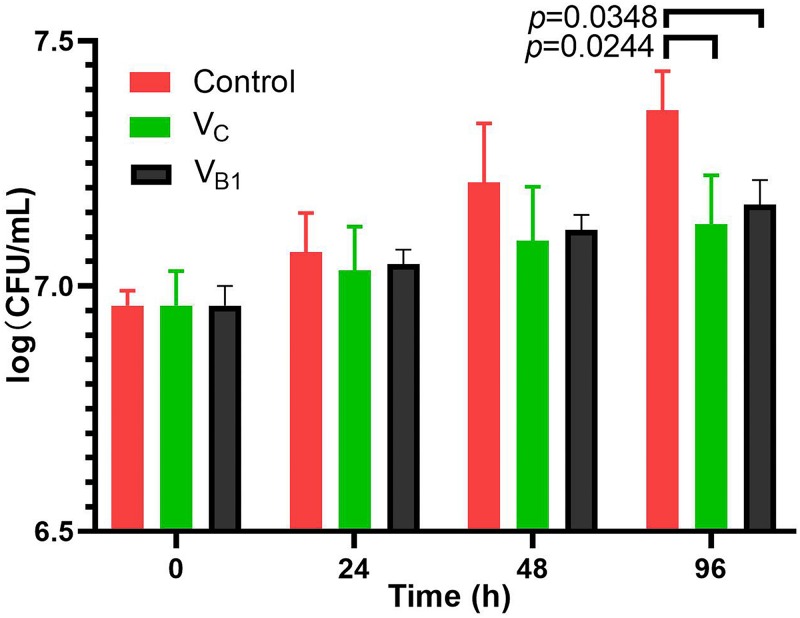
CFU assays of BCG treated with V_B__1_(8 mM) and Vc (5 mM).

Since the CFU counting of BCG is similar after V_B__1_ (8 mM) and V_C_ (5 mM) treatment for 48 h and the differentiation continues comparing with the control group (Figure 1), all the omics studies were performed with final concentration of 8 mM for V_B__1_ and 5 mM for Vc. SMRT sequencing was used to determine the genome-wide distribution of methylated bases in V_C_-treated, V_B__1_-treated and control BCG. The average sequencing coverage was 363-531X (Supplementary Table 1). Analysis of the SMRT-sequenced data detected *N*^6^-methyl-adenine (m6A) in the three samples, whereas m4C sites, 5,317, were only detected in the Vc-treated BCG sample (Supplementary Table 2).

### Distribution of m6A Loci

In total, 5,191 m6A sites were detected in the three BCG samples and 4,641 of them (89.4%) were common to all three, with 19, 20, and 11 unique sites in V_B__1_- treated, V_C_-treated and control BCG samples, respectively (Supplementary Figure 1). Five m6A sequence motifs were detected (Supplementary Table 3). The CTCCAG/CTGGAG motif represented in approximately 74% of them (1,931) and was distributed randomly across the genome. A total of 3,756 adenines in this motif (97.3%) were methylated in the three BCG samples, suggesting that this motif has a protective role. A total of 1,784 protein coding sequences (CDS) were affected by m6A, accounting for 42.6% of BCG genes. Enrichment analysis revealed that important pathways involved in metabolism, cellular processes and environmental information processing were significantly affected by m6A (FDR < 0.05, Supplementary Table 4). COG class enrichment analysis also revealed that m6A was present in genes participating in cell envelope biogenesis and outer membrane, also suggesting that the m6A modification has a protective role. In contrast, un-methylated genes (i.e., those that did not have m6A or m4C modifications) were enriched in pathways Ribosome and Translation, Membrane transport, and Metabolism of cofactors and vitamins (Supplementary Table 5). COG class enrichment analysis also revealed abundant un-methylated genes with unknown biological function.

### Distribution of m4C Loci

Surprisingly, m4C was observed only in samples treated with V_C_, with 5,317 m4C sites in three motifs (Supplementary Table 3). 2,374 CDSs were affected by m4C, accounting for 56.7% of BCG genes. Among these, 1,221 CDSs were also methylated at adenine sites (i.e., 69.1% of the 1,767 m6A modified genes). Enrichment analysis revealed that m4C modification mainly affected genes involved in amino acid metabolism, cell envelope biogenesis and outer membrane (FDR < 0.05, Supplementary Table 6). This suggests that, since V_C_ treatment hampers BCG growth, genes involved in cell envelope biogenesis become more protected.

### Presence of m6A and m4C in Intergenic Regions (IGRs)

**FIGURE 2:**
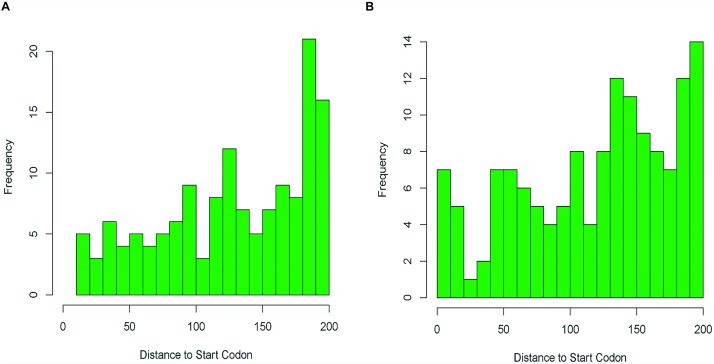
Distances relative to the start codon in intergenic region of m6A sites **(A)** and m4C sites **(B)** basing on the methylation sites in V_B__1_ and V_C_ treated BCG samples.

In eukaryotes, promoter methylation has been suggested to inhibit gene expression. Our analysis discovered 4,781 m6A sites located in CDSs, and 410 (7.9%) in IGRs. Since the promoter regions in bacteria are usually located within 100 bp upstream of the start codon (Heyden et al., 1975; Chen et al., 2010), we calculated the distance between the methylation sites and the downstream start codon. Only m6A modifications located within 200 bp upstream of the start codon were assumed to affect genes. In this way, only 143 out of the 410 m6A sites in IGRs probably affected the promoters of 138 genes (Supplementary Table 12). In BCG treated with V_C_, 423 m4C were located in IGRs, where 142 affected promoters of 140 genes (Supplementary Table 12). Further analysis revealed a relative enrichment of m6A at positions in the range −180 to −200 bp (Figure 2A) that was less evident for the m4C sites (Figure 2B).

### The Transcriptome of BCG

**FIGURE 3:**
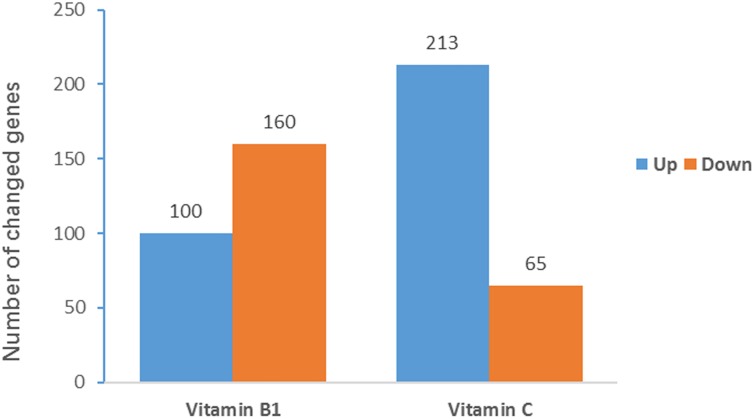
Significantly regulated BCG genes after vitamin treatment. The *Y*-axis indicates the gene number of significantly changed genes. Partial omics data of V_B__1_ can be found in this published paper (Song et al., 2019).

To investigate how V_B__1_ and V_C_ treatment affects BCG growth, RNA-seq was used to compare the transcriptome of the three replicates of each BCG sample (V_B__1_-treated, V_C_-treated and control). The mean FPKM (Fragments Per Kilo bases per Million reads) was 274.4 for V_B__1_-treated BCG (282.5 for control) and 289.4 for Vc-treated BCG (281.9 for control). A total of 260 genes were regulated in V_B__1_-treated BCG, and 278 in Vc-treated BCG (Figure 3). Of these, more down-regulated genes were found for V_B__1_-treated BCG, which were enriched in pathways like Nitrogen metabolism and Two-component system (Supplementary Table 7). In contrast, V_C_-treated BCG showed more up-regulated genes involved in replication and repair. In the Vc-treated BCG samples, lower expression was found in genes involved in energy metabolism. In addition, we note that expression of genes involved in biosynthesis of siderophore group non-ribosomal peptides was inhibited by V_B__1_, but stimulated by V_C_ (Supplementary Table 7). The same pattern was observed for another six genes, with two of them (mycobactin polyketide synthetase gene *MbtB* and *MbtC*) participating in this pathway.

### DNA Methylation and RNA Expression

To investigate the relationship between gene methylation and gene expression, we analyzed the methylation changes in significantly regulated genes. There were 19 unique m6A sites in V_B__1_-treated BCG, but only one of them was located within the coding region of significantly down-regulated genes, away from the promoter region. For the 20 unique m6A sites in V_C_-treated BCG, two were located in the promoter region of significantly up-regulated genes (Supplementary Table 11). In that sample, m4C was found in the coding region of 31 down-regulated genes (47.7%) and 138 up-regulated genes (64.8%) (Supplementary Table 11), but it was observed in the promoter region only in 11 up-regulated genes (Supplementary Table 12). These results suggest that gene expression is not affected when methylation is in the coding region, but when m4C is located in the promoter region it contributes to up-regulate BCG gene expression. To validate this hypothesis, we compared the gene expression levels of methylated and non-methylated genes, based on their FPKM. Although no significant changes were observed for m6A-modified genes (Supplementary Figure 2), the expression levels of 65 genes harboring m4C sites in the promoter region was significantly higher than in the control that had no m4C sites (mean FPKM = 272.3 and 222.9, respectively and *p* < 0.05) (see Supplementary Figure 3).

### Metabolite Analysis

**FIGURE 4:**
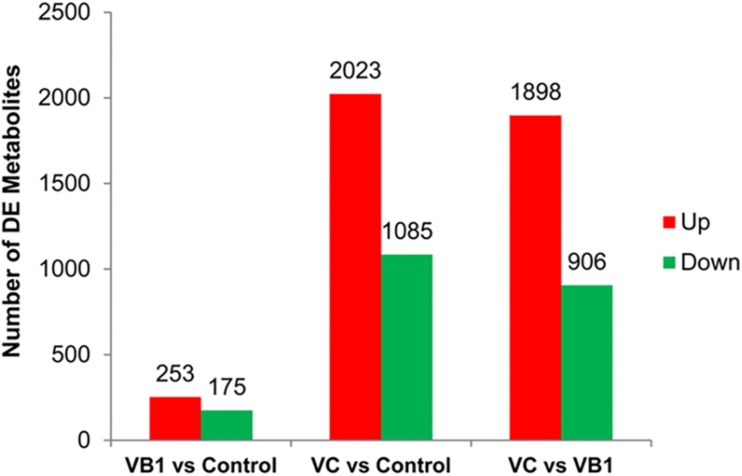
The diagram for differential metabolites found in the three BCG samples examined. Partial omics data of V_B__1_ can be found in this published paper (Song et al., 2019).

Liquid chromatography-mass spectrometry (LC-MS) was used to analyze six bacterial sediments of each of the three BCG samples (V_B__1_-treated, V_C_-treated and control). After V_B__1_ or V_C_ treatment, the number of differential metabolites was 428 and 3,208, respectively (Figure 4).

**FIGURE 5:**
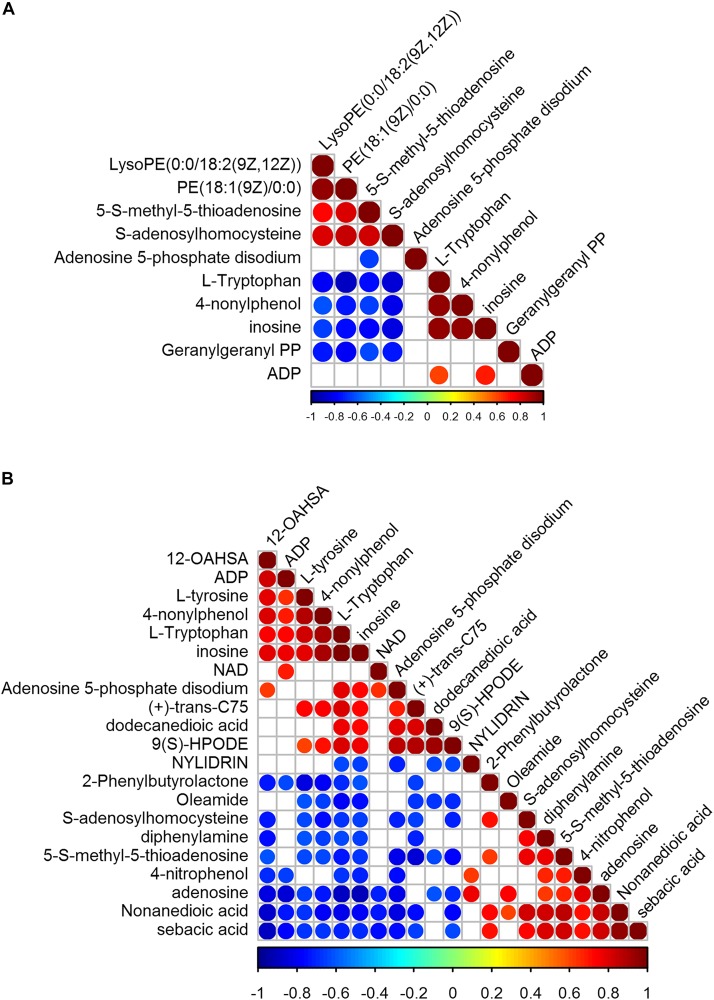
Correlation of significantly variant metabolites of BCG treated with V_B__1_ **(A)** and BCG treated with Vc **(B)** relative to control. Partial omics data of V_B__1_ can be found in this published paper (Song et al., 2019).

We identified 41 metabolites with significant variation, 10 in V_B__1_-treated BCG and 21 in Vc-treated BCG (Figure 5). In V_B__1_ treated BCG samples, the concentration of LysoPE[0:0/18:2(9Z,12Z)], PE[18:1(9Z)/0:0], 5-*S*-methyl-5-thioadenosine and *S*-adenosylhomocysteine increased, but the concentration of Adenosine 5-phosphate disodium, L-Tryptophan, 4-nonylphenol, inosine, Geranylgeranyl PP and ADP decreased. In Vc treated BCG samples, the concentration of Nylidrin, 2-Phenylbutyrolactone, Oleamide, *S*-adenosylhomocysteine, diphenylamine, 5-*S*-methyl-5-thioadenosine, 4-nitrophenol, adenosine, Nonanedioic acid and sebacic acid increased, in the contrast, the concentration of 12-OAHSA, ADP, L-tyrosine, 4-nonylphenol, L-Tryptophan, inosine, NAD, Adenosine 5-phosphate disodium, (+)-*trans*-C75, dodecanedioic acid and 9(S)-HPODE decreased (Figure 5). Enrichment analysis revealed that variant metabolites were mainly clustered in amino acid metabolism pathways (Supplementary Table 8). After V_B__1_ treatment, variant metabolites were significantly enriched in cysteine and methionine metabolism (Supplementary Table 7).

### Metabolism of Amino Acids

Since exposure to vitamins led to variant metabolites enrichment in amino acid metabolism pathways, we performed additional amino acids content analysis using six bacterial sediments of each of the three BCG samples (V_B__1_-treated, V_C_-treated and control). Exposure to vitamins significantly reduced (V_B__1_) or increased (V_C_) amino acid content (Supplementary Table 9). The expression changes associated with amino acid metabolism were also analyzed, as described below.

### V_B__1_ Addition

After V_B__1_ addition to BCG, a key enzyme involved in thiamine (V_B__1_) metabolism (hydroxymethylpyrimidine/phosphomethylpyrimidine kinase [2.7.4.7]) was significantly down-regulated (Supplementary Figure 4), suggesting that V_B__1_ addition reduced the requirement for *de novo* synthesis of thiamine. Another significantly down-regulated gene was chorismate mutase [5.4.99.5] (Supplementary Figure 5 and Supplementary Table 10), which catalyzes the transformation from chorismate to prephenate, the precursor of phenylalanine and tyrosine. This is consistent with the observed 80% reduction in Phe and Tyr after V_B__1_ addition. This treatment also caused almost 50% reduction in alanine, although the gene expression of alanine dehydrogenase [1.4.1.1] showed no significant down-regulation. Instead, the gene expression of the two alanine dehydrogenase genes (*JTY_RS14370* and *JTY_RS14375*) increased slightly, which suggests that alanine reduction was caused by lower availability of pyruvate.

We then examined the pyruvate metabolism and glycolysis pathways. In the pyruvate metabolism pathway, no significant up-regulation was observed, indicating that the conversion of pyruvate to other metabolites did not increase. However, in the glycolysis pathway, a 51-fold reduced expression was observed for 6-phosphofructokinase 2 [2.7.1.11], an enzyme that converts fructose-6P into fructose-1,6P2, which may explain the 50% reduction in alanine after V_B__1_ addition (Supplementary Figure 5). For histidine synthesis, although no significant gene expression change was observed, genes involved in five out of nine steps were down-regulated. Genes involved in the synthesis of other amino acids also showed the same pattern, where at least one gene encoding a key enzyme was down-regulated (Supplementary Table 10).

### V_C_ Addition

V_C_ addition caused ornithine to increase 1.6-fold (Supplementary Table 9), consistent with a significant up-regulation of genes involved in three out of five steps required to transform glutamate to ornithine (Supplementary Figure 6). Also, three out of four genes encoding enzymes that convert ornithine to arginine doubled their expression levels. V_C_ addition also increased the content of aspartate 2.7-fold. L-asparaginase [3.5.1.1], which converts Asn to Asp, increased 2-fold after V_C_ addition (Supplementary Table 10). Thus, the observed increase of Thr might be due to an increase in Asp content (Supplementary Figure 6) and to a down-regulation of genes degrading Thr (Supplementary Table 10). The tryptophan synthase [4.2.1.20] gene was also significantly down-regulated (Supplementary Figure 6), consistent with the reduction of Trp in these conditions.

Strikingly, the cysteine synthase A [2.5.1.47] gene was significantly up-regulated in BCG treated with either V_B__1_ or Vc (Supplementary Figures 5, 6). Finally, we found no direct association between base modification in coding or promoter regions and expression of genes involved in amino acid metabolism (Supplementary Table 10). Taken together, we found significant regulation of several genes directly involved in amino acid biosynthesis and metabolism, consistent with amino acid content changes. However, most gene expression changes were not significant. We speculate that treatment with V_B__1_ and V_C_ changes amino acid levels by altering precursor concentrations.

### Cysteine Inhibits BCG Growth

**FIGURE 6:**
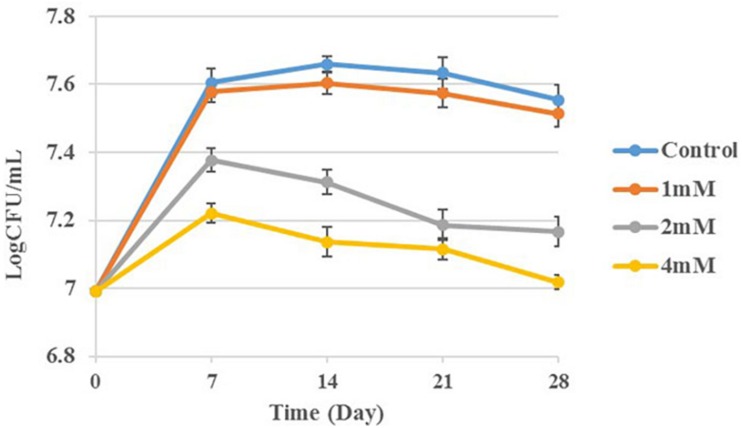
BCG treatment with cysteine at concentrations 0, 1, 2, and 4 mM. Aliquots taken at indicated times and plated to determine CFUs.

Following the observation that vitamins treatment increase the transcription levels of cysteine synthase A, we determined the effect of cysteine on BCG replication. Different concentrations of cysteine (0, 1, 2, and 4 mM) were used for CFU calculation and the results are summarized in Figure 6. The growth curve demonstrates that cysteine inhibits BCG growth in a concentration-dependent manner.

## Discussion

Vitamins such as biotin and thiamine are essential for *M. tuberculosis* growth and infection (Tyagi et al., 2017), and Mtb scavenges these vitamins from the host. At the same time, supplementation of some vitamins together with anti-TB drugs has been shown to improve chemotherapy outcomes (Vilcheze et al., 2018). Clinical trials have shown that Vc supplementation improves the healing process in tuberculosis patients (Tjandrawinata et al., 2018), although no clinical improvement (sputum conversion) of patients was observed when using vitamin D (V_*D*_). Therefore, the mechanism by which some vitamins can ammeliorate TB requires further investigation.

Mtb growth is inhibited by vitamins A (V_*A*_) and V_*D*_ (Greenstein et al., 2012, 2014), and recent studies have shown that V_B__1_ and Vc also significantly inhibit BCG growth (Pei et al., 2019; Song et al., 2019). Thus, due to the potential supplementary role of certain vitamins in TB treatment, analysis of the BCG methylome can help elucidate the relationship between base methylation, gene expression and metabolic products.

Many DNA methylation enzymes are part of RM systems, and are involved in the defense against phages and viruses. We found 42.6% of BCG genes were m6A methylated, and these were enriched in key metabolism and cellular processes, hinting that essential genes in BCG are always protected by methylation.

Regulation of gene expression by DNA methylation occurs via more than one mechanism. DNA methylation hinders the interaction between DNA and regulatory proteins by direct atomic steric effects (Sanchez-Romero et al., 2015), or indirectly by altering DNA structure itself; this reduces the thermodynamic stability of the double helix, resulting in alteration of gene expression (Sanchez-Romero et al., 2015). Additionally, some DNA-binding proteins inhibit methylation of specific DNA sequences by binding to unmethylated DNA with high affinity.

In general, positive or negative transcription regulation depends on differences in the location of methylation sites in the promoter region, modulation of regulatory proteins, or differences in binding between regulatory proteins and unmethylated/methylated DNA target sequences. In the present study, we found that 138 genes (64.8%) harboring m4C in the coding region, and 11 genes harboring m4C in the promoter region, were up-regulated in Vc-treated BCG. Additionally, 65 genes that harbored m4C sites in the promoter region showed expression levels significantly higher than the control (no m4C sites), based on FPKM values. This suggests that m4C can promote gene transcription to some extent, but using mechanisms that are still unknown. The m4C modification was observed in type I R-M systems, suggesting that, in addition to m6A modifications, they can also use this modification for host protection (Morgan et al., 2016).

Recent studies have shown that N^4^-cytosine DNA methylation can regulate transcription and pathogenesis in *Helicobacter pylori* (Kumar et al., 2018). Indeed, in human gastric adenocarcinoma cells, *H. pylori* mutant with increasing m4C methylation increased adhesion, while the bacteria without m4C methyltransferase halved the adhesion rate and significantly reduced *H. pylori*-induced apoptosis. Additionally, in *Cyanobacterium Synechocystis* sp. PCC 6803m4C, methylation is involved in the regulation of gene expression, fine-tuning of DNA replication and DNA repair mechanisms (Gartner et al., 2019). Until now, however, the role of m4c modification in Mtb has been uncertain.

In our study, we found 11 up-regulated genes harboring an m4c modification in the promoter region (Supplementary Table 12) of BCG genome are homologous with these genes which had been studied to show important roles in Mtb. For example, Rv2416c (JTY_RS12525) is a secretory protein that enhances survival inside macrophages (Ganaie et al., 2011). Rv3130c (JTY_RS16210) encodes a triacylglycerol synthase that synthesizes triacylglycerol. This is proposed as an energy source during dormancy, and its disruption prevents triacylglycerol accumulation under inducing conditions (Sirakova et al., 2006). Rv2626c (JTY_RS13655) plays a significant role in macrophage pro-inflammatory response and in mycobacterial survival during infection (Sun et al., 2017). Rv0256c (JTY_RS01360), which can be used as a potential marker for the serodiagnosis of tuberculosis patients, may inhibit nitric oxide (NO) production in activated macrophages (Bhat et al., 2013; Abraham et al., 2014). Rv1187 (JTY_RS06330), encoding pyrroline-5-carboxylate dehydrogenase, was shown to be involved in the proline-utilization pathway in Mtb, and this pathway could be a valuable therapeutic target against TB (Jena et al., 2015). Additionally, Rv2308 (JTY_RS11970), Rv2100 (JTY_RS10875) and Rv2015c (JTY_RS10425) encoding hypothetical proteins, Rv2618 (JTY_RS13615) encoding transcription regulator, Rv2893 (JTY_RS14970) encoding oxidoreductase, Rv3892c (JTY_RS20265) encoding PPE protein are also methylated at the promoter region.

In metabolomics, we found that the concentrations of several metabolites including tryptophan, inosine, NAD and ADP decreased after V_B__1_ and Vc treatment. Tryptophan is classified as an essential amino acid in humans and must be acquired through the diet. This requirement alleviates the concern of common targets within humans. Recent studies found that tryptophan synthase which catalyzes the final step in tryptophan biosynthesis is one potentially novel anti-tubercular drug target (Abrahams et al., 2017). In addition, treatment with 6-FABA 2-amino-6-fluoro-benzoic acid (6-FABA) chemically induces Mtb tryptophan auxotrophy. Together with immune- mediated trypothan starvation, this results in mycobacterial death. The results provide genetic and chemical validation of the tryptophan biosynthesis pathway as a target for highly active antibiotics (Zhang et al., 2013).

Inosine is made up of deaminizing adenosine and can be released extracellularly under inflammatory conditions (Jabs et al., 1995; Schmidt et al., 1995). Inosine has been reported to exert nutritional and neuroprotective functions in nerve cells, and stimulates mast cell degranulation by activating adenosine A3 receptor (Jin et al., 1997). Moreover, inosine exhibits anti-inflammatory activity by inhibiting the release of inflammatory cytokine from activated T cells and promoting the formation of IL-10. Clinical trials have proposed that the use of inosine to raise serum urate levels may have benefits for at least some multiple sclerosis patients. The effect of this treatment is likely to be a consequence of inactivation of peroxynitrite-dependent free radicals (Markowitz et al., 2009).

NAD(P) is an indispensable cofactor for all organisms and its biosynthetic pathways are proposed as promising novel antibiotics targets against pathogens such as Mtb. It is reported that NAD(P) play the import role in immune response, gene silencing, DNA repair, regulation, redox reaction, energy and cellular metabolism which participate in more than 300 biochemical reactions, amounting to 17% of all the classical reaction (You, 1985; Bi et al., 2011). Nicotinamide adenine dinucleotide (NAD+) is essential for a variety of organisms, and it is both a coenzyme for hydride-transfer enzymes and a substrate for NAD+-consuming enzymes, such as ADP-ribose transferases, poly(ADP-ribose) polymerases, cADP-ribose synthases, and sirtuins.

Since the synthesis pathways of these significantly reduced metabolites are mostly drug targets for the treatment of tuberculosis, we hypothesized that the addition of vitamins may directly or indirectly affect the synthesis or metabolic processes of these products. This could potentially inhibit bacterial growth.

A striking result in this study is that V_B__1_ treatment of BCG resulted in content reduction of almost all types of amino acids, whereas Vc treatment caused the opposite effect. Through RNA-seq analysis, we found that the gene encoding cysteine synthase A was significantly up-regulated after vitamin treatment, suggesting that Cys synthesis is promoted. It has been reported that isoniazid (INH)/Cys combination potentiates the killing of Mtb by stimulating respiration of Mtb from non-respiring dormancy (Vilcheze et al., 2017). High levels of intracellular cysteine can induce ROS production, causing DNA damage and oxidation of cysteine to cystine in *E. coli* (Park and Imlay, 2003). Indeed, transition metals, such as copper or iron, catalyze the oxidation of cysteine into cystine, leading to the production of hydrogen peroxide (H_2_O_2_), superoxide, and hydroxyl radicals (Kachur et al., 1999). This high concentration of cysteine could therefore initiate cationic stress, resulting in the formation of ROS and DNA damage (Imlay et al., 1988; Demple and Harrison, 1994).

In our study, the gene encoding cysteine synthase A was significantly up-regulated after V_B__1_ and Vc treatment and this hinting that the synthesis of cysteine is promoted. In addition, *in vitro* growth test show that cysteine inhibit the growth of BCG in a concentration-dependent manner. Therefore, we hypothesize that V_B__1_ and Vc inhibit BCG growth by increasing the concentration of cysteine, which can eventually damage DNA via Fenton reaction. This hypothesis is consistent with the transcriptomics results, where Vc induced the expression of genes related to DNA repair, suggesting that Vc may indirectly affect DNA integrity.

**FIGURE 7:**
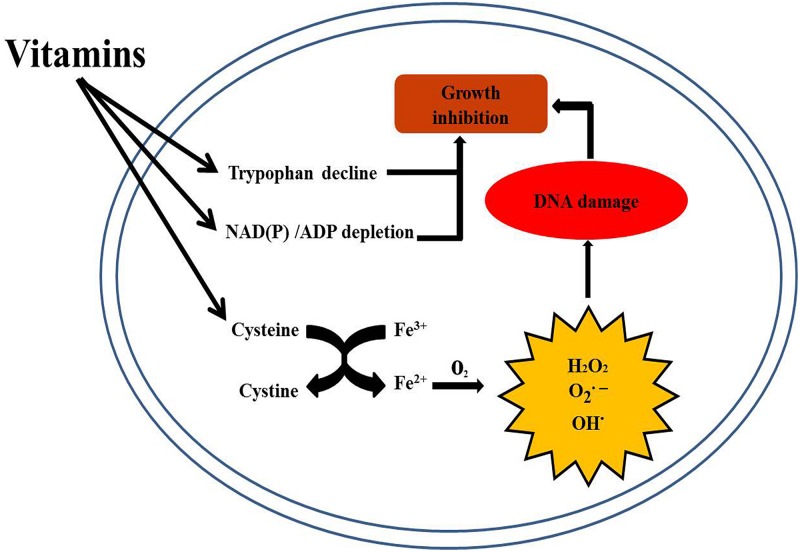
Schematic representation of the mechanism of action of vitamins against BCG growth.

Notably, the scientists found that Vc sterilized Mtb cultures via Fenton reaction and increased iron concentration, which correlates with the bactericidal activity of V_C_ (Vilcheze et al., 2013). The results shown in the present study suggest that BCG growth inhibition may not be due just to high levels of iron, but also to cysteine. In addition, decreased concentrations of tryptophan, NAD(P) and other metabolites are also potential reasons for vitamin inhibition of BCG growth (Figure 7). Our study also identifies another possible mechanism by which vitamins inhibit BCG growth. Therefore, V_B__1_ and Vc supplementation may enhance the action of anti-tuberculosis drugs.

## Data Availability Statement

The datasets generated for this study can be found in the Gene Expression Omnibus, accession numbers GSE114949 and GSE141513.

## Author Contributions

NS designed this study and wrote the manuscript. YZ analyzed the data and prepared the figures. YC, ML, YT, ZC, and GD participated in the data analysis. HZ participated in the data analysis and contributed to the writing. SL contributed to the concepts and the writing. All authors have read and approved the final manuscript.

## Conflict of Interest

The authors declare that the research was conducted in the absence of any commercial or financial relationships that could be construed as a potential conflict of interest.

## Footnotes

1 http://www.fruitfly.org/seq_tools/promoter.html

2 http://genome2d.molgenrug.nl/g2d_pepper_promoters.php
