# Supplementary material for: Vitamin B and Vitamin C Affect DNA Methylation and Amino Acid Metabolism in Mycobacterium bovis BCG
Source: Front Microbiol. 2020 Apr 22;11:812. doi: 10.3389/fmicb.2020.00812 (PMC7188828; doi:10.3389/fmicb.2020.00812)
Supplement: Supplementary file 1 [file Image_1.PDF]

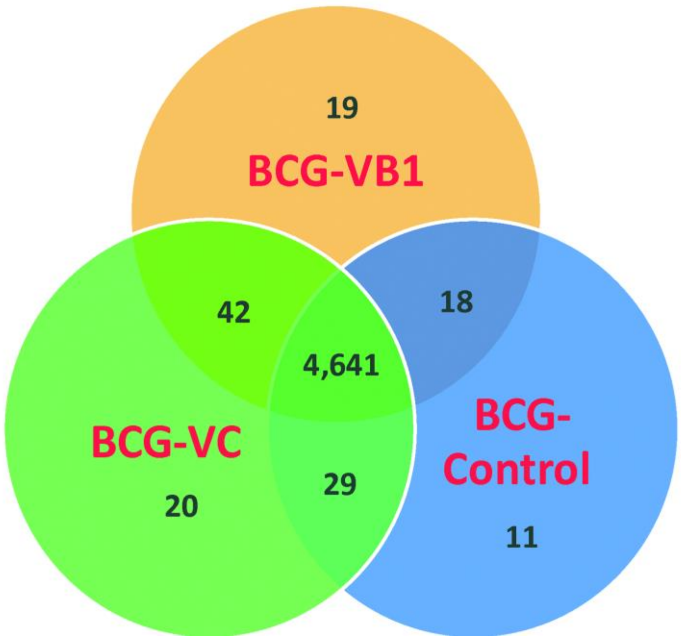

Supplementary Figure 1. Distribution of the number of m6A sites among the three BCG samples studied.

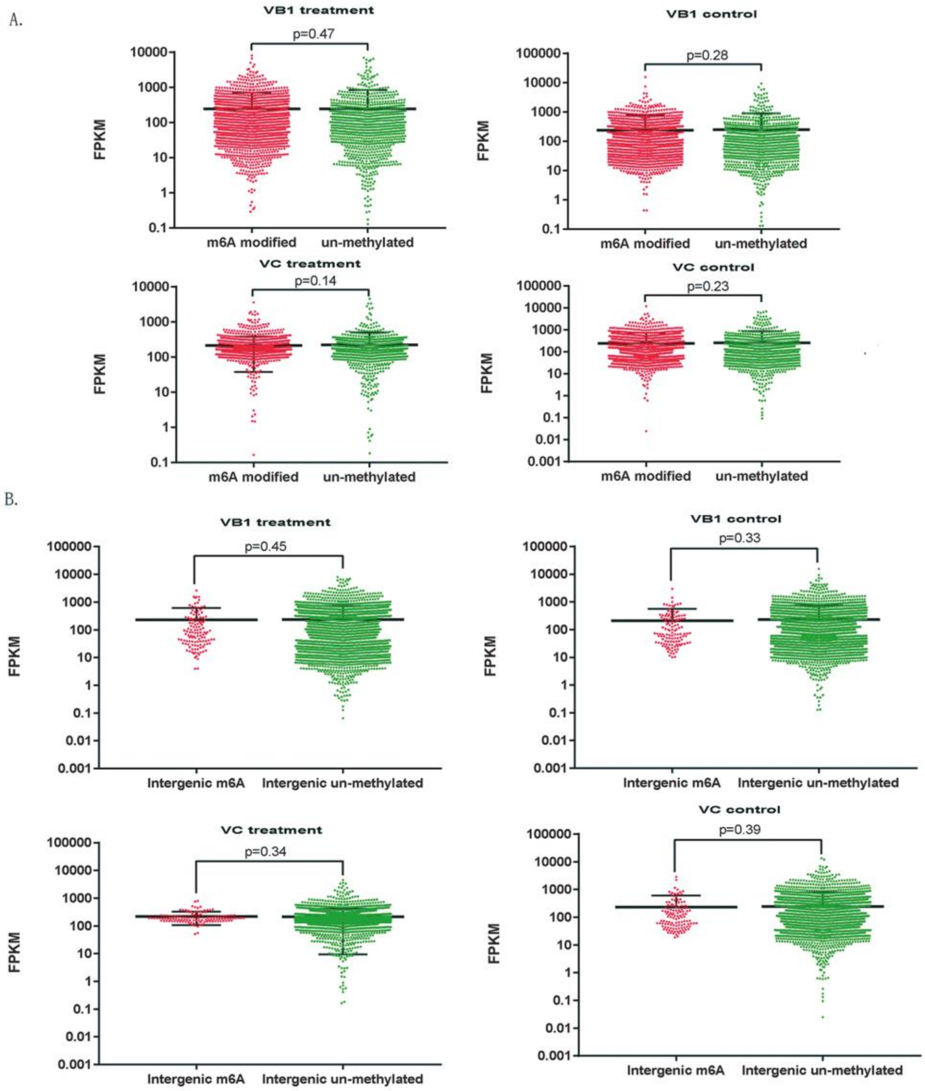

Supplementary Figure 2: Comparison of gene expression level (FPKM) between m6A- modified genes and m6A-unmethylated genes. (A). The expression level (FPKM) of genes whose coding region was modified by m6A (red plots) and genes whose coding region having no m6A modification (green plots). (B). The expression level (FPKM) between genes whose promoter was modified by m6A (red plots) and genes whose promoter having no m6A modification (green plots). The P value calculated by PRISM indicates that the gene expression level between the two groups had no significant changes. Whether the BCG sample had been treated by Vitamins (V_{B1} treatment, V_C treatment) or not (V_{B1} control, V_C control) was labeled on the top of each picture.

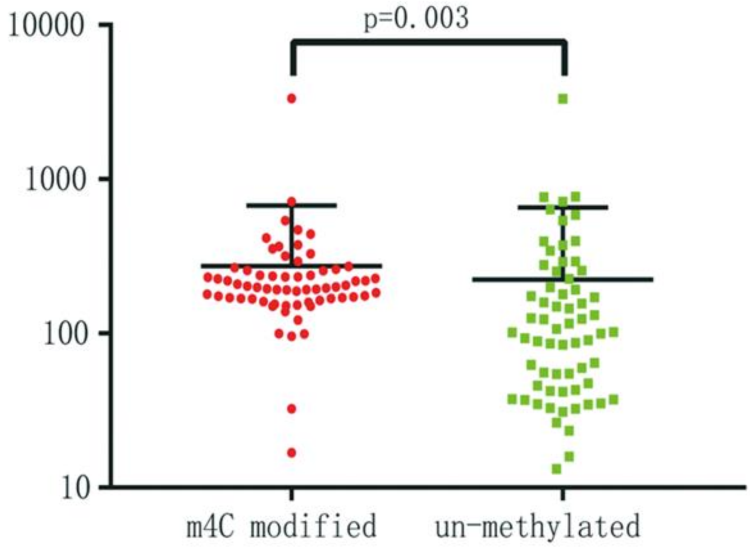

Supplementary Figure 3. Gene expression comparison between genes with or without m4C modifications in the promoter region.

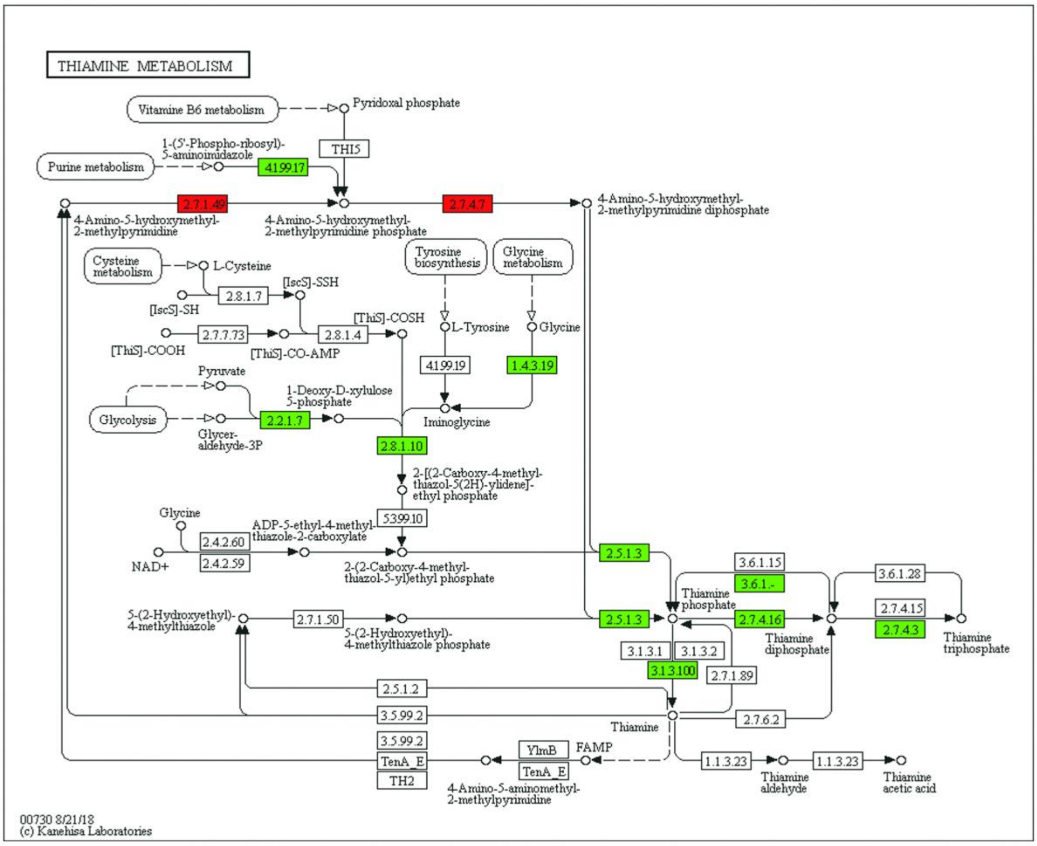

Supplementary Figure 4. Thiamine (V_{B1}) metabolism pathway of BCG after V_{B1} addition: significantly down-regulated genes (red) and genes with no significant regulation (green).

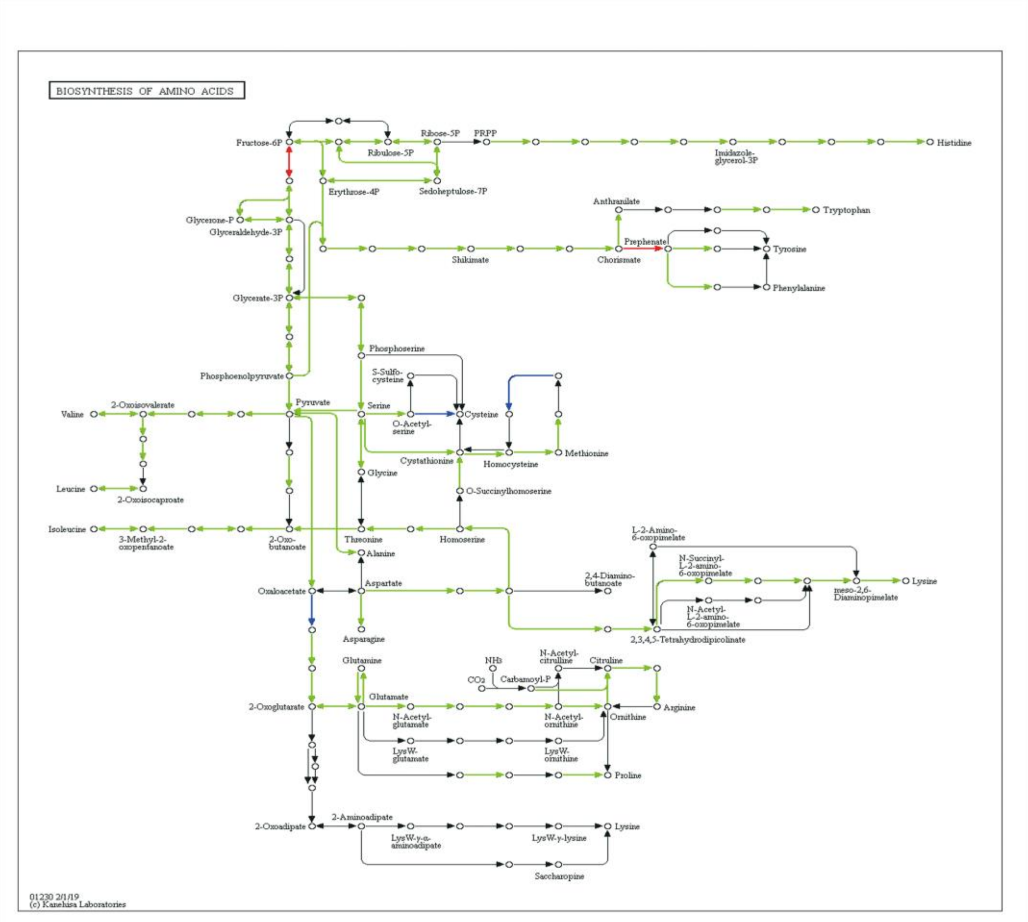

Supplementary Figure 5. Amino acid synthesis schematic diagram of BCG after V_{B1} addition: significantly down-regulated genes (red lines), significantly up-regulated genes (blue lines) and genes with no significant regulation (green lines).

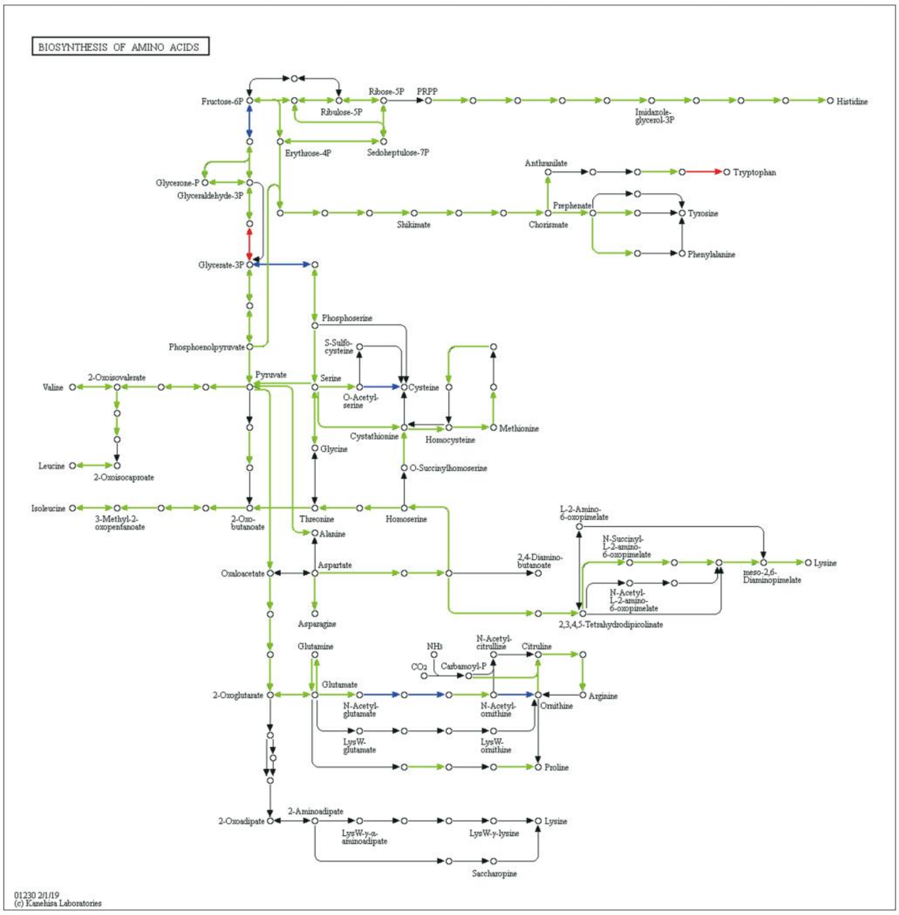

Supplementary Figure 6. Amino acid synthesis diagram of BCG after V_C addition: significantly down-regulated genes (red lines), significantly up-regulated genes (blue lines) and genes with no significant regulation (green lines).
